# Gender Dynamics in Vaccine Acceptance and Hesitancy Among Primary Caregivers in Ethiopia: A Mixed-Methods Study

**DOI:** 10.3390/vaccines13100998

**Published:** 2025-09-24

**Authors:** Geteneh Moges Assefa, Michael Tarekegn, Kasahun Negash, Betibebu Mulugeta, Sintayehu Abebe, Baye Denekew, Mhret Ayele, Azmeraw A. Tesfahun, Gedamu Kassie, Virginia Stulz, Makida Berhan, Muluken Desalegne Muluneh

**Affiliations:** 1 Amref Health Africa in Ethiopia, P.O. Box 20855, Addis Ababa 1000, Ethiopia; michael.tarekegn@amref.org (M.T.); kasahun.negash@amref.org (K.N.); betibebu.mulugeta@amref.org (B.M.); sintayehu.abebe@amref.org (S.A.); baye.denekew@amref.org (B.D.); mihret.ayele@amref.org (M.A.); makida.berhan@amref.org (M.B.); mulusef@yahoo.com (M.D.M.); 2 Development Management, Debre Berhan University, Debre Berhan P.O. Box 445, Ethiopia; azmeraw2008@gmail.com; 3 School of Public Health, Bahir Dar University, Bahir Dar P.O. Box 79, Ethiopia; gedamukassie@gmail.com; 4 School of Nursing and Midwifery, University of Newcastle, Callaghan, NSW 2308, Australia; virginia.stulz@newcastle.edu.au

**Keywords:** vaccine acceptance, hesitancy, gender dynamics: caregivers, immunization

## Abstract

Background/Objectives: Vaccination uptake in Ethiopia is deeply shaped by gender norms, with women serving as primary caregivers but often limited by low autonomy, while men typically control household decisions but remain less engaged in child health. This study examines gendered influences on vaccine hesitancy and acceptance to inform future strategies. Methods: A community-based cross-sectional mixed-methods study was conducted in four regions of Ethiopia (Amhara, Oromia, Afar, and Tigray). Quantitative data were collected from 992 caregivers through multistage stratified sampling procedure, whereas qualitative data were collected from 26 in-depth interviews, 24 exit interviews and 11 key informant interviews and were analyzed thematically guided by the WHO Behavioural and Social Drivers framework. Multivariable logistic regression was conducted to determine the associations. Results: The result highlighted that the proportion of caregivers who reported willingness to vaccinate their child with all recommended vaccines was high (93.5%) and more likely among those with higher education, family support, religious support, and frequency of health worker contact. However, 51.1% of caregivers displayed some degree of vaccine hesitancy, with higher prevalence among males. Hesitancy was linked to traditional beliefs and norms that assign vaccination responsibility to mothers, urban residence, and being employed, while secondary education, family support, and religious support were protective. While acceptance is high, hesitancy persists. Gender roles, education, social support, and health worker engagement influence immunization outcomes. Conclusions: The study highlighted that expanding immunization across all age groups and reducing persistent hesitancy requires a shift toward gender-responsive strategies including integrating a gender perspective into the immunization programs that address traditional norms and misinformation.

## 1. Introduction

Vaccination is a transformative and cost-effective public health strategy, preventing an estimated 4.4 million deaths annually and significantly reducing the burden of vaccine-preventable diseases, particularly among children under five [1]. Achieving herd immunity depends on high vaccine acceptance, defined as the willingness of individuals especially caregivers to adhere to recommended immunization schedules [2,3]. Each year, more than 20 million infants globally miss at least one dose of routine vaccines, and over 13 million remain “zero-dose” children, never having received any basic immunizations [4]. These gaps result from both supply-side challenges, including poverty, remote locations, and insecurity, and demand-side barriers such as vaccine hesitancy, defined by WHO as the delay or refusal of vaccination despite availability [5]. Hesitancy, driven by trust in vaccines, complacency, accessibility, is further exacerbated by misinformation, cultural beliefs, and systemic distrust [5,6,7,8,9]. In Ethiopia, childhood immunization coverage is estimated to rise from 14.3% in 2000 to projections estimating 53.6% by 2025 (Projection) still far below the 90% target set by the Immunization Agenda 2030 (IA2030) [10,11]. National strategies such as the Expanded Program on Immunization (EPI) and the Health Extension Program (HEP) have improved access, particularly in rural areas, yet disparities persist among pastoralist, conflict-affected, and hard-to-reach populations [12,13]. Ethiopia’s Health Sector Transformation Plan II and EPI Multi-Year Plan prioritize universal access and community engagement, but challenges remain in addressing hesitancy and achieving equity [14].

Gender plays a critical but underexplored role in immunization outcomes. In sub-Saharan Africa, including Ethiopia, women are typically the primary caregivers, responsible for managing children’s health, including vaccinations [15]. Their knowledge and access to health services are vital, yet social norms, economic dependence, low decision-making autonomy, and domestic burdens often limit their ability to act [16]. Conversely, men, who frequently control household finances and key decisions, have less direct involvement in child health services and are more susceptible to misinformation through peer networks and social media [17]. Gender norms shape caregivers’ ability to act and limit autonomy, while also affecting health worker (both female and male) capacities and institutional frameworks. Both caregivers and female health extension workers face distinct gender-related barriers, from limited mobility and decision-making power to policy-level oversights that hinder uptake [13,18,19].

Evidence highlighted that male involvement and spousal support enhance vaccine uptake, while reliance solely on maternal responsibility leads to missed or delayed immunizations [20,21,22]. These dynamics are particularly relevant in Ethiopia, where vaccine hesitancy often manifests as delayed uptake rather than outright refusal, frequently due to competing caregiving demands [23]. While maternal education and antenatal care attendance consistently correlate with higher vaccine uptake, male partner engagement and intra-household decision-making remain under-addressed in Ethiopian health policies [12,13,24]. In response, global strategies such as WHO’s Immunization Agenda 2030 and the Behavioural and Social Drivers (BeSD) of Vaccination framework call for inclusive, equitable immunization systems. They emphasize gender-responsive programming that considers how gender norms influence vaccine access, confidence, and uptake [11,25]. Similarly, Gavi’s Gender Equality and Immunization Framework highlights the need to empower women while engaging men and dismantling structural barriers to under-vaccination [26]. Despite growing recognition of gender’s influence, substantial evidence gaps persist in Ethiopia. Most national datasets lack gender-disaggregated insights into vaccine confidence, decision-making authority, and social support systems. Few studies explore how gender intersects with structural barriers such as geography, poverty, and conflict. Existing research often relies on self-reported data and fails to capture the emotional, cognitive, and contextual dimensions of vaccine behavior, limiting the development of gender-responsive interventions. While global frameworks like WHO’s Immunization Agenda 2030 and Gavi’s Gender Equality Framework emphasize gender-responsive programming, implementation in Ethiopia has primarily focused on maternal engagement, overlooking opportunities to involve men and address intra-household dynamics.

Therefore, this study aims to address these gaps by examining gender dynamics in vaccine hesitancy and acceptance among caregivers in four Ethiopian regions: Amhara, Oromia, Afar, and Tigray.

To better conceptualize the complex interplay between gender and vaccine behaviors, this study employs a gender-responsive adaptation of the WHO BeSD framework. This approach explicitly considers how gender norms, roles, and power dynamics influence cognitive, emotional, behavioral, and trust-related drivers of vaccine acceptance and hesitancy. This conceptualization guides both the quantitative and qualitative analyses, enabling a nuanced understanding of gendered vaccine dynamics in Ethiopia.

## 2. Materials and Methods

### 2.1. Study Design and Area

A community-based cross-sectional study design was employed to explore gender differences in vaccine-related outcomes among caregivers, using quantitative data supplemented by qualitative data to enrich understanding and contextualize findings. The study was conducted across four regions of Ethiopia, Amhara, Oromia, Afar, and Tigray, covering a total of ten woredas (both rural and urban kebeles (The lowest level of government in Ethiopia’s administrative structure)). These study sites were selected based on immunization coverage, demographics representations and geographic accessibility.

### 2.2. Statistical Analysis

#### 2.2.1. Population, Sample Size, and Sampling Techniques

For the quantitative data, this study targeted caregivers of under five children eligible for immunization in the project intervention areas. The sample size was calculated using an OpenEpi single population proportion formula. Among several potential indicators considered for this study, the national immunization coverage among under-five children in Ethiopia between 2000 and 2019, estimated at 51.1%, was used as the basis for the sample size calculation, as it provides the maximum sample size to ensure adequate statistical power [27]. This resulted in a final sample size of 992 participants. A multistage stratified sampling technique was employed to select 992 study participants. Four intervention regions were purposively selected, followed by a random selection of ten districts. Finally, systematic random sampling was applied to select households with eligible children in each kebele, using proportional allocation.

For the qualitative data, 26 in-depth interviews (IDIs), 24 exit interviews and 11 key informant interviews (KIIs) participants were purposively selected from each study district included in the quantitative study, based on their involvement in the EPI service. KII participants consisted of health office officials, exit interview participants consisted of primary caregivers, interviewed during their exit from vaccination services, while IDIs were conducted with health care providers at the facility level and community leaders.

#### 2.2.2. Variables

This study assessed two binary outcome variables: vaccine acceptance and vaccine hesitancy (both yes/no). Independent variables included sociodemographic factors (sex, age, education, residence, number of under-five children, marital and employment status) and social and health system support factors (family and religious support, health worker recommendation and contact, knowledge of immunization sites, and attitudes toward women’s vaccination responsibility).

#### 2.2.3. Operational Definition

Vaccine hesitancy: A composite vaccine hesitancy index (range: 0–1) was constructed from 15 indicators across four dimensions: behavioral (4 items), cognitive (6 items), emotional (3 items), and trust-related (2 items). Items were normalized and aggregated into a continuous score. Based on the sample median (0.339), participants were categorized as hesitant (>0.339) or non-hesitant (≤0.339), in line with WHO SAGE guidance and methods used in prior studies [5,28].

Vaccine acceptance: Defined as the proportion of caregivers who reported willingness to vaccinate their child with all recommended vaccines [25].

Knowledge about immunization: Caregivers’ knowledge was assessed using eight belief-based items. Each item was scored as 1 for correct responses and 0 for incorrect or “don’t know” responses. Negatively phrased items were reverse-coded. Total scores ranged from 0 to 8 and were categorized using modified Bloom’s cut-off points: poor (0–2), moderate (3–5), and good (6–8) knowledge levels [29].

Attitudes toward immunization: Attitudes were measured using six Likert-scale statements (1 = strongly disagree to 5 = strongly agree). To quantify attitudes, responses were numerically coded, and negatively worded items were reverse coded to maintain consistency in scoring direction. A total attitude score was then calculated by summing the coded responses across all statements.

Scores were classified using Bloom’s taxonomy as follows: negative attitude (≤60% of maximum score), neutral attitude (61–79%), and positive attitude (≥80%) [30]. Using a modified Bloom’s taxonomy framework, the total scores were categorized into three levels: negative attitude (scores ≤ 60% of the maximum possible score), neutral attitude (scores between 61% and 79%), and positive attitude (scores ≥ 80%).

#### 2.2.4. Data Collection Tools and Procedures

Data were collected using a mixed-methods approach. Quantitative data were obtained via a pre-tested, structured interviewer-administered questionnaire with categorical and Likert-scale items covering knowledge (8 items), attitudes (6 items), vaccine hesitancy (15 items), vaccine acceptance (1 item), vaccine confidence (3 items), social support (5 items), access to immunization services (2 items), satisfaction (1 item), and gender-related decision-making (2 items). The questionnaire items were adapted from multiple validated sources, including the WHO SAGE report, BeSD tools, and the Parent Attitudes About Childhood Vaccines (PACV) survey [5,25,31]. The survey was conducted among primary caregivers using mobile data collection platforms (ODK/KOBO) for real-time electronic entry and quality assurance [32]. Qualitative data were collected through key informant and in-depth interviews using semi-structured guides aligned with the BeSD framework.

#### 2.2.5. Data Quality Control

Data quality was ensured through close supervision and routine monitoring during data collection. For the quantitative component, supervisors and the data manager conducted daily checks of completed questionnaires to ensure completeness and internal consistency. Global Positioning System-enabled tools verified that data were collected from the intended households, and random back-checks by field supervisors further strengthened data accuracy. A booster session was held mid-fieldwork to reinforce data collection protocols. After validation, data was uploaded to a secure server for additional checks. The internal consistency of the 15-item vaccine hesitancy scale was assessed using Cronbach’s alpha yielding an acceptable reliability coefficient (α = 0.73). Prior to analysis, exploratory factor analysis (EFA) was conducted to assess the underlying factor structure, confirming the multidimensionality of the scale with acceptable factor loadings (>0.4) across items. Pilot testing with 50 caregivers ensured clarity and cultural relevance of items. These validation steps support the robustness of the hesitancy measure used in this study. Data triangulation across multiple sources enhanced the validity and reliability of the results.

#### 2.2.6. Analysis Methods

Data collected via Kobo mobile applications were extracted, cleaned, and verified by the data manager before analysis. A total of 12 filled questionnaires were excluded from the analysis due to response inconsistency related issues. Data analysis was undertaken on the 980 caregivers’ responses. The Quantitative data were analyzed using STATA software. Descriptive statistics were generated to summarize participant characteristics and key study variables, and bivariable logistic regression was conducted to examine associations between vaccine acceptance, vaccine hesitancy, and potential predictor variables. Variables with *p*-values less than 0.25 in bivariable analyses were included in multivariable logistic regression models to identify independent predictors.

For vaccine acceptance, the final multivariable model included education level, marital status, family and religious support, health worker recommendation and contact, knowledge of immunization sites, and attitudes toward women’s responsibility in child vaccination.

The model showed good fit (Hosmer–Lemeshow χ^2^ = 5.77, *p* = 0.673) and no multicollinearity (mean VIF = 1.50). Similarly, the multivariable model for vaccine hesitancy retained ten variables, including sex, residence, education level, employment status, marital status, family and religious support, health worker recommendation and contact, and attitudes toward women’s vaccination responsibility. Interaction terms for sex by education and family support by health worker recommendation were tested. The model fit was adequate (Hosmer–Lemeshow χ^2^ = 6.53, *p* = 0.589), with no evidence of multicollinearity (mean VIF = 1.46). Qualitative findings were analyzed thematically and used to complement and contextualize the quantitative results, providing deeper insight into caregivers’ perceptions, social influences, and barriers related to childhood vaccination acceptance and hesitancy.

## 3. Results

### 3.1. Socio-Demographic Characteristics

Out of 980 caregivers surveyed (across Amhara, Oromia, Afar, and Tigray), 65.8% were aged 18–34, and 92.2% were married. Women constituted 65% of participants. Education levels varied: 18.9% were illiterate, 34.6% had primary education, and 24.3% had education beyond secondary school. About 71.8% of respondents were unemployed, and 54.6% identified as Muslim. The average household had three children, and about 62.7% of caregivers reported one child under five (see Table 1).

### 3.2. Knowledge About Immunization

**Figure 1:**
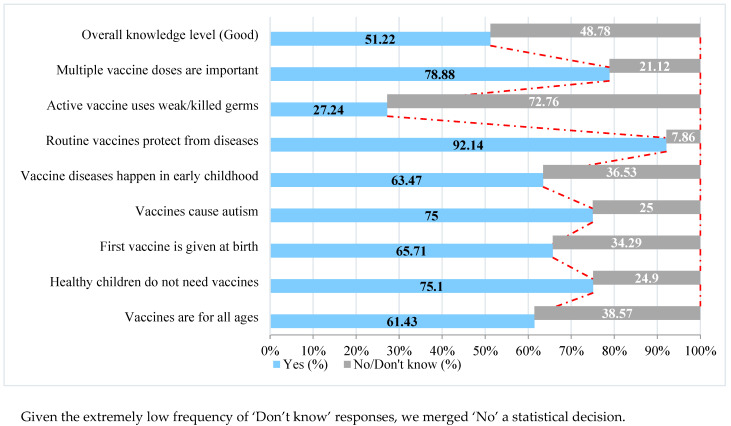
Overall and item-specific knowledge levels on immunization, June 2025.

The mean vaccine knowledge score was 5.39 (out of eight) (±1.43), indicating moderate to good knowledge with 502 (51.2%) demonstrating good knowledge, 441 (45.0%) moderate, and 37 (3.8%) poor knowledge. Awareness was particularly high for statements affirming vaccine importance and safety (75.1% knew healthy children need vaccination; 75% rejected autism myths). Gender differences were minimal (Figure 1).

### 3.3. Attitudes and Confidence Toward Immunization

The study assessed attitudes toward vaccination using six key statements, with responses recorded on a five-point Likert scale. A total attitude score was then calculated by summing the coded responses across all statements and categorized into three levels (negative, neutral and positive attitude using a modified Bloom’s taxonomy framework (see operational definition part).

**Figure 2:**
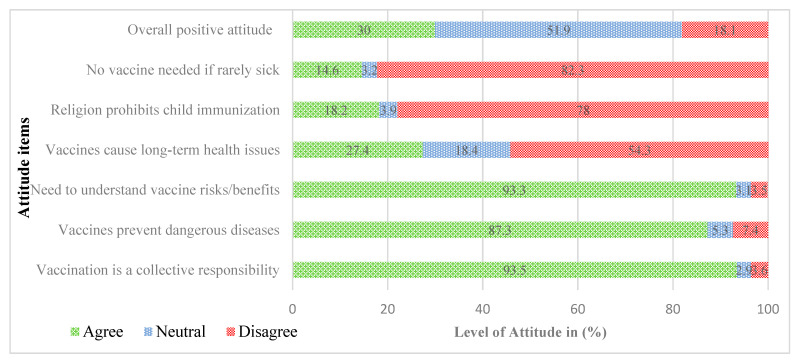
Overall and item-specific attitude towards immunization, June 2025.

The results showed that about 30% expressed a positive attitude toward vaccines; 52% were neutral and 18% had a negative attitude toward vaccines. Female caregivers showed more favorable attitudes than male caregivers towards immunization. Neutrality was higher among males (58.3%) than females (48.5%). A strong majority agreed that vaccination is a collective responsibility (93.5%), prevents dangerous diseases (87.3%), and that understanding vaccine risks and benefits is essential (93.3%). Fewer respondents agreed that vaccines cause long-term health issues (27.4%), that religion prohibits child immunization18.2%) and that vaccination is unnecessary for rarely sick children (14.6%) (See Figure 2).

**Figure 3:**
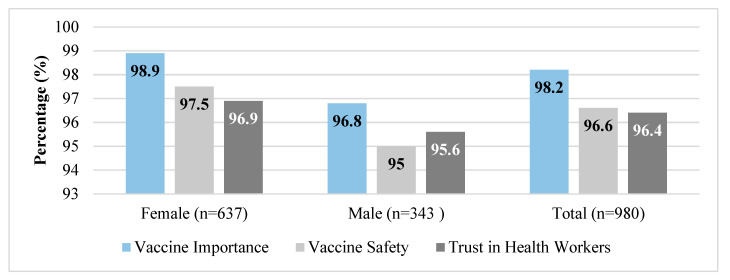
Overall confidence in vaccine importance, safety, and trust in health workers, disaggregated by sex, June 2025.

Vaccine confidence was found to be very high among the surveyed caregivers A vast majority (98.2%) believed vaccines are important, 96.6% considered them safe, and 96.4% trusted health workers. Disaggregated by sex, female caregivers consistently reported slightly higher confidence levels than males (see Figure 3).

### 3.4. Social Support and Health System Engagement in Childhood Vaccination

**Figure 4:**
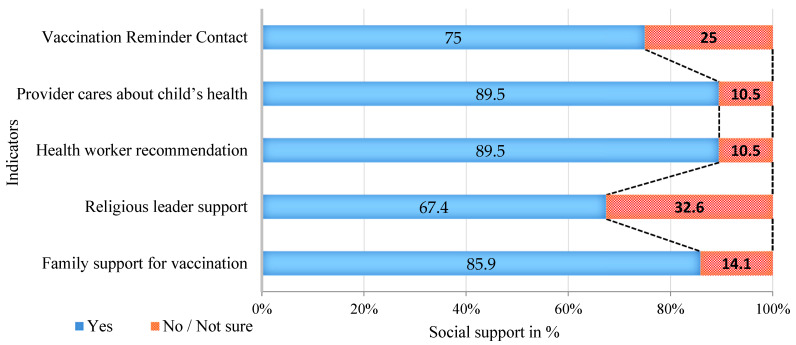
Caregivers Reporting Social Support and Health System Engagement, June 2025.

**Figure 5:**
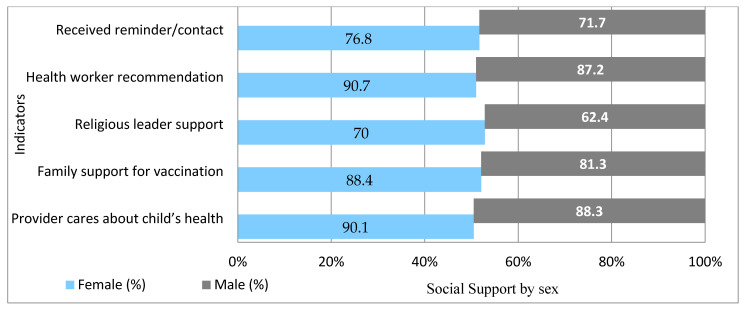
Caregivers Reporting Social Support and Health System Engagement by Sex, June 2025.

Strong support networks were evident: 86% received family support, 67.4% felt supported by religious leaders, and 89.5% received recommendations from health workers. Women consistently reported higher support across all domains. (See Figure 4 and Figure 5).

### 3.5. Gender Roles and Decision-Making

**Figure 6:**
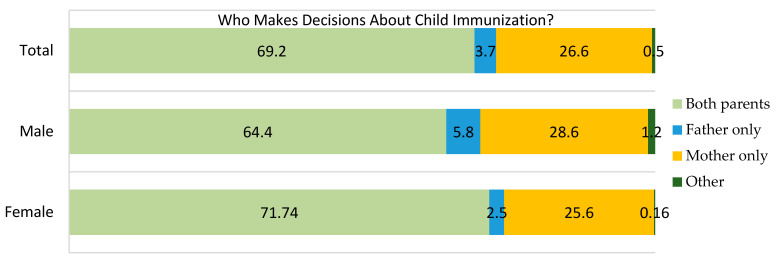
Distribution of Decision-Making Roles in Child Immunization by Sex, June 2025.

Although 678 (69.2%) reported joint decision-making for vaccination, a significant number (65.6%) still perceived immunization as the mother’s primary responsibility. Only 4.4% of households reported mothers solely deciding on finances, with a higher proportion among females (*n* = 457, 71.7%) than males (*n* = 221, 64.4%). A considerable percentage of males (*n* = 98, (28.6%) and female (*n* = 163, 25.6%) caregivers reported that immunization related issues are solely decided by mothers, suggesting skewed gender role in households (Figure 6).

**Figure 7:**
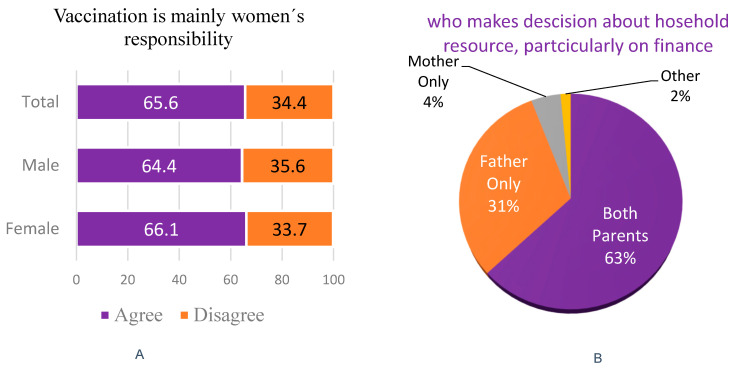
Attitudes on child vaccination as primarily the mother’s responsibility and who makes decision about household resources, particularly on finance, June 2025.

Two-thirds (65.6%) of caregivers stated that ensuring child vaccination is primarily the mother’s responsibility, with more females (*n* = 432, 68.1%) than males (*n* = 211, 61.5%) agreeing. This suggests limited male participation in the practical aspects of child health, despite their reported role in decision-making (Figure 7A). Regarding household resource decisions, about 63.9% of caregivers indicated joint decision-making by both parents, similar among females (*n* = 402, 63.1%) and males (*n* = 224, 65.3%). Fathers alone made decisions in 31.0% of cases, while mothers were sole decision-makers in a minority (*n* = 43, 4.4%) (Figure 7B) of cases.

### 3.6. Access and Satisfaction with Vaccination Services

Most caregivers found vaccination services accessible and affordable, with over 95% reporting satisfaction. Barriers were minor but include vaccine stock-outs, delayed openings, and long wait (see Table 2).

### 3.7. Vaccine Acceptance

**Figure 8:**
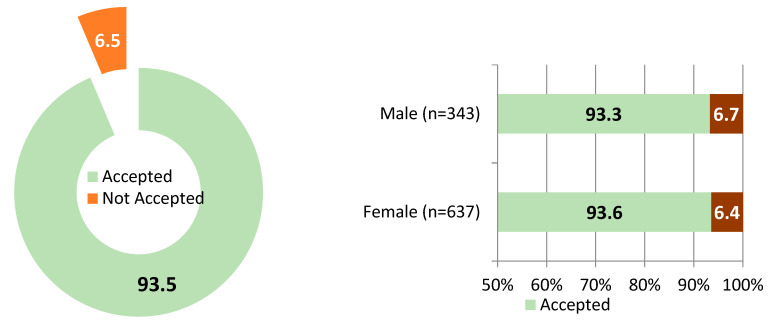
Overall vaccine acceptance and disaggregation by gender, June 2025.

Acceptance was high at 93.5% (95% CI: 91.7–94.9%), with gender difference. Most non-accepting caregivers expressed intent to complete vaccination, indicating low outright refusal (Figure 8).

Qualitative findings strongly reinforced these quantitative patterns, illustrating that the decision to vaccinate was deeply rooted in caregivers lived experiences and values. Across all four study regions, caregivers described vaccination not simply as a medical task but as a profound parental duty, often shaped by prior experiences with child illness or health improvements linked to immunization. In Oromia, one caregiver explained how her perception of vaccination was informed by the positive health outcomes observed in her children. One of the participants shared (P1):

“Since my first child was fully vaccinated, we rarely visit the health institution for sickness. That is why I never miss the appointments for the younger ones.”

This comment reflects a common view that immunization leads to fewer illnesses and reduced health care costs, reinforcing parental commitment to completing vaccine schedules.

Similarly, in Tigray, a mother described her unwavering determination:

Another participant shared (P2): “No matter how early or how far, I carry my child for every dose; it is on me to protect her.”

Such expressions of responsibility and agency were consistently reported, suggesting that vaccine acceptance is driven more by proactive health-seeking behavior than by compliance alone. This motivation extended even into areas affected by conflict. In Tigray, where health services had been interrupted, providers and caregivers made concerted efforts to resume immunization. One health worker shared (P3):

“After the war, we started zero dose of Penta in the form of campaign, and we have completed three doses now. The trend is backing to the right track.”

This example illustrates the resilience of both the community and health system, with vaccination quickly rebounding as a priority despite the disruption. For many, the ability to resume child immunization was a signal of recovery and hope.

### 3.8. Predictors of Vaccine Acceptance

Engagement with health workers was another important predictor. Caregivers who had recent contact with health professionals were twice as likely to vaccinate their children (AOR = 2.01; 95% CI: 1.07–3.76). Qualitative interviews highlighted that health providers not only delivered information but acted as mobilizers and facilitators, especially in pastoralist and remote communities. A health worker in Afar noted that pastoralist families often pause vaccinations during seasonal migration, requiring outreach teams to follow and provide services. This illustrates that missed vaccinations are often due to logistical barriers—such as distance, mobility, and system limitations—rather than outright refusal.

Despite high motivation levels, structural and contextual barriers persist in some of the study areas related to insecurity and conflict, particularly in Amhara and Tigray regions. A health official in Amhara explained that conflict disrupted service delivery, participant (P6) stating, “The primary obstacles are instability and conflicts… health services operate amid war and instability.” Infrastructure weaknesses further limited access; a health worker in Mille (Afar) described challenges in meeting demand with only one health center serving the entire town. Additionally, supply-side issues, such as vaccine shortages post-conflict, have eroded trust. A caregiver in Tigray expressed skepticism about vaccine quality following war-induced stockouts, participant (P4) saying, “After the war, the health institution ran out of vaccines; now I doubt the quality of the new supply.” These concerns underscore how historical disruptions continue to affect current perceptions of vaccine reliability and safety.

Quantitative analysis revealed several significant predictors of vaccine acceptance among caregivers. Educational attainment was a strong and consistent factor: individuals with primary (AOR = 0.48; 95% CI: 0.32–0.72), secondary (AOR = 0.33; 95% CI: 0.21–0.52), and above-secondary education (AOR = 0.47; 95% CI: 0.29–0.79) were significantly more likely to accept vaccination compared to those without formal education. This trend was supported by qualitative findings, where more educated parents demonstrated proactive health-seeking behaviors and a better understanding of vaccines’ preventive benefits.

Social and community support also emerged as powerful enablers. Caregivers who received support from family were 56% less likely to refuse vaccination (AOR = 0.44; 95% CI: 0.28–0.70), while those supported by religious leaders were 42% less likely (AOR = 0.58; 95% CI: 0.42–0.80). Interviews confirmed these associations; for instance, a religious leader in Amhara emphasized the harmony between faith and science, Participant (P7) stating, “We keep telling parents that science and faith agree vaccine save life; so, bring your children.” In Afar, community elders played a critical role in countering misinformation, with one elder noting efforts to correct misconceptions among young fathers who feared vaccines might weaken boys. These testimonies highlight how trusted local figures, religious and cultural, have been instrumental in sustaining community-wide vaccine confidence and combating misinformation.

Engagement with health workers was another important predictor. Caregivers who had recent contact with health professionals were twice as likely to vaccinate their children (AOR = 2.01; 95% CI: 1.07–3.76). Qualitative interviews highlighted that health providers not only delivered information but acted as mobilizers and facilitators, especially in pastoralist and remote communities. A health worker in Afar noted that pastoralist families often paused vaccinations during seasonal migration, requiring outreach teams to follow and provide services. This illustrates that missed vaccinations are often due to logistical barriers—such as distance, mobility, and system limitations—rather than outright refusal.

A caregiver in Tigray expressed skepticism about vaccine quality following war-induced stockouts, participant (P4) saying, “After the war, the health institution ran out of vaccines; now I doubt the quality of the new supply.” The accompanying multivariable logistic regression analysis (Table 3) quantifies these associations and supports the qualitative insights. Statistically significant factors include higher education levels, family and religious support, and health worker contact, all of which positively correlate with vaccine acceptance. Conversely, factors like marital status, knowing where to immunize, and gender-related attitudes showed less definitive associations. These results emphasize the multifaceted nature of vaccine acceptance, shaped by educational, social, and systemic influences.

### 3.9. Vaccine Hesitancy: Composite Index

**Figure 9:**
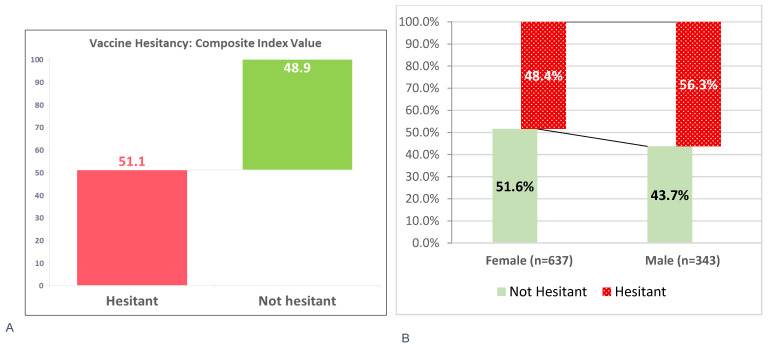
Overall Vaccine Hesitancy status (**A**) and hesitancy disaggregated by Sex (**B**), June 2025.

The composite index of vaccine hesitancy revealed that 501 caregivers (51.1%; 95% CI: 47.99–54.25%) were hesitant, with higher rates among males (56.3%) than females (48.4%). Conversely, 479 caregivers (48.9%) were not hesitant, including more females (51.6%) than males (43.7%) (see Figure 9). However, when asked directly, 765 caregivers (78.1%) denied any hesitancy, suggesting a disconnect between perceived and actual hesitancy—possibly due to limited awareness or social desirability bias. Many caregivers equate hesitancy only with outright refusal, overlooking doubts or delays.

Health workers reported low hesitancy toward familiar vaccines like measles and polio, seen as routine and trusted. As one EPI officer in Oromia noted, “People trust them.” In Amhara, a health provider remarked that rapid deployment led many to believe the vaccine lacked sufficient testing. Conflict-affected areas like Tigray saw added layers of distrust, where caregivers questioned the origin and safety of vaccines distributed amid political instability. As one provider explained (P8), “Some parents doubt the safety… They ask where the vaccine came from and hesitate even though we explain it is safe.”

These findings underscore the nuanced nature of vaccine hesitancy: often less about refusal and more about uncertainty, shaped by familiarity, context, and trust in systems.

These narratives illustrate the complexity behind the composite hesitancy index, where caregivers may be categorized as hesitant due to doubts or delayed uptake even when direct questioning finds no outright refusal.

**Figure 10:**
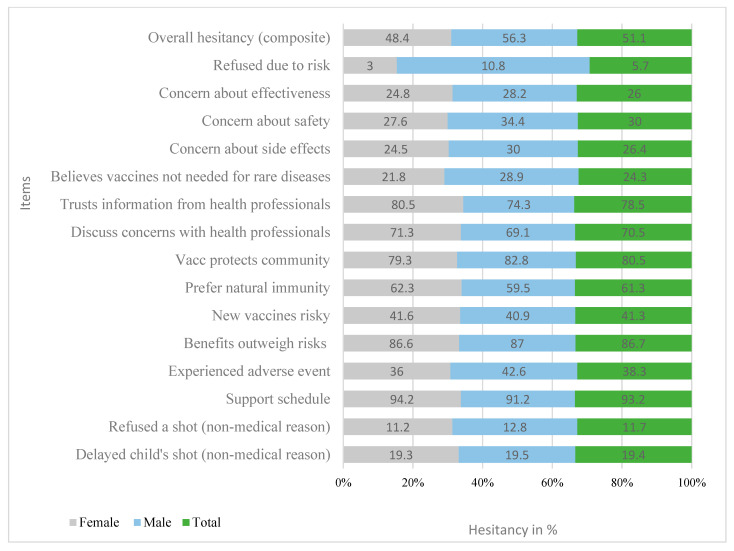
Individual Item Responses on Vaccine Hesitancy Among respondents, June 2025.

Of the 980 caregivers surveyed, 501 (51.1%) showed some level of vaccine hesitancy. Specifically, 190 (19.4%) delayed vaccination—similar across genders—and 115 (11.7%) reported outright refusal. Despite this, the majority (93.2%) supported the recommended immunization schedule, and 86.7% believed the benefits of vaccines outweigh the risks. However, 38.3% reported adverse events, and safety concerns persisted: 18% were somewhat concerned and 8.5% very concerned about side effects. Notably, 41.3% felt new vaccines were riskier, and 29.7% preferred natural immunity. Trust remained relatively high—78.5% trusted health professionals and 70.5% felt comfortable discussing concerns—while 87.8% agreed vaccination helps protect others. Still, 24.3% believed vaccines were unnecessary for rare diseases, and 5.7% reported refusing vaccines specifically due to safety fears, with male caregivers reporting higher rates of concern-driven refusal (See Figure 10).

### 3.10. Predictors of Vaccine Hesitancy

Multivariable analysis identified five key factors significantly associated with vaccine hesitancy. Agreement with traditional gender norms—specifically, the belief that vaccination is solely a woman’s responsibility—increased hesitancy by 69% (AOR = 1.69; 95% CI: 1.25–2.28; *p* = 0.001). Qualitative data showed that while many mothers internalized this duty and felt a strong sense of personal responsibility, the lack of shared support from partners or family often led to delays or missed appointments, particularly in rural and pastoralist communities. One caregiver in Amhara expressed, “Vaccination is part of what I must do as a mother… I want the same [health] for my baby.”

Education had a protective effect: caregivers with secondary education were 47% less likely to be hesitant (AOR = 0.53; 95% CI: 0.31–0.90; *p* = 0.019). However, qualitative insights revealed that a minority of formally educated individuals, particularly in Amhara and Oromia, were more vocal in spreading vaccine misinformation—such as conspiracy theories involving microchips or infertility—underscoring that partial or misapplied knowledge can also contribute to hesitancy. As one provider in Oromia noted, “Some educated people were the ones spreading rumours.”

Urban residence was linked to a 62% increase in hesitancy (AOR = 1.62; 95% CI: 1.21–2.16; *p* = 0.001). Caregivers in urban areas were more exposed to misinformation through social media and informal networks, leading to doubts about vaccine safety and campaign motives. In contrast, rural communities showed higher trust in health workers and lower exposure to misinformation. A provider in Amhara shared, “In town, there were people saying the COVID vaccine has a chip or causes infertility.”

Employment status also influenced hesitancy. Unemployed caregivers had 41% lower odds of hesitancy (AOR = 0.59; 95% CI: 0.40–0.88; *p* = 0.009). (see Table 4) Employed individuals often faced scheduling conflicts, especially when male household members were unavailable to assist with transport or decision-making. In regions like Afar and Oromia, such logistical barriers were compounded in pastoralist or migratory households. Qualitative data linked this to time constraints and rigid work schedules, limiting their participation in immunization activities. In addition, employed caregivers are more exposed to rumors and misinformation via social media and peer networks, fostering fears about vaccine safety and mistrust.

Conversely, social support emerged as a strong protective factor. Family encouragement reduced hesitancy by 75% (AOR = 0.25; 95% CI: 0.09–0.71; *p* = 0.009), and support from religious leaders lowered it by 50% (AOR = 0.50; 95% CI: 0.36–0.71; *p* < 0.001) (see Table 4). Health providers in Afar and Tigray emphasized the role of religious and community leaders in dispelling myths and fostering trust, particularly during immunization campaigns. Family members, especially spouses and mothers-in-law, were also crucial in building caregiver confidence. In areas affected by recent outbreaks or vaccine-preventable deaths, shared grief catalyzed stronger vaccine demand, as one official in Afar noted: “When families lose a child to measles, it changes everything.”

No statistically significant differences were found across regions in relation to vaccine acceptance; therefore, results are presented without regional stratification.

Overall, the findings highlight that while education and social support can reduce hesitancy, structural barriers, traditional gender roles, and misinformation—particularly in urban and employed populations—remain key challenges. These insights suggest targeted strategies for increasing vaccine confidence, including gender-inclusive outreach, support mobilization, and countering misinformation in urban settings.

## 4. Discussion

This mixed-methods study investigated gender differences in vaccine acceptance and hesitancy among 980 caregivers across four Ethiopian regions. The findings reveal how gender intersects with social, cultural, and health system factors to shape immunization behaviors. Although overall vaccine acceptance was high, underlying hesitancy patterns showed distinct gendered attitudes and decision-making roles, highlighting the urgent need for gender-sensitive approaches to improve vaccine equity.

The study confirmed a remarkably high overall vaccine acceptance rate of 93.5%, with similar levels among female (93.6%) and male (93.3%) caregivers. This aligns with regional and global findings: a study in Addis Ababa, Ethiopia reported 96% caregiver acceptance [23], while research from Rwanda and Uganda also highlights strong maternal trust and motivation toward immunization [21,33,34]. A systematic review of 42 studies across Africa further supports this, showing widespread caregiver willingness despite structural barriers [16]. These findings corroborate the strong caregiver acceptance rates reported in our study. These findings reflect Ethiopia’s robust primary health care system, strong community trust in health workers, and effective communication through the Health Extension Program. Confidence in vaccine importance (98.2%), safety (96.6%), and trust in health workers (96.4%) was also notably high, reinforcing a strong immunization culture. However, qualitative insights revealed key gender dynamics: women took proactive responsibility for childhood immunization, while men were more passive, consistent with patterns observed in Nigeria and Uganda [21,35]. These gendered roles suggest that maternal engagement is central to vaccine uptake, but greater male involvement may require targeted strategies.

Building on these high levels of acceptance, the study further explored key predictors that shape caregivers’ decisions to vaccinate. One of the strongest factors identified was educational attainment. Caregivers with primary, secondary, or higher education were more likely to vaccinate their children. This aligns with evidence from various African contexts, where education enhances health literacy and informed decision-making [33,36,37,38]. However, studies also show that strong community engagement, such as outreach by health workers, can significantly improve vaccination uptake even among caregivers with little or no formal education, demonstrating the capacity of community health systems to overcome literacy-related barriers [39].

A gendered pattern also emerged in relation to education: educated mothers actively sought vaccination services, while educated fathers often played passive roles or, in some cases, contributed to misinformation. This highlights that education alone is not enough and must be paired with gender-sensitive health communication. Caregivers who received encouragement from family members or religious leaders were more likely to vaccinate their children. Women reported higher levels of such support than men, reflecting gender norms around caregiving roles. This finding aligns with studies from Nigeria and Uganda, where community and faith-based networks have been shown to shape health-seeking behaviors and support maternal roles in child immunization. For example, in Uganda, Babirye et al. (2011) found that mothers, as primary decision-makers for immunization, were strongly influenced by community encouragement [21]. In Nigeria, Oku et al. (2017) demonstrated that engaging religious leaders in communication strategies enhanced vaccine uptake [40]. Melillo et al. (2022) further confirmed that in many low- and middle-income countries, trusted religious actors help expand immunization coverage by building trust and addressing community concerns [41].

Qualitative findings from this study also pointed to the influence of male-led community groups in dispelling harmful beliefs, such as the notion that vaccines weaken boys. However, men’s limited engagement in these networks remains a missed opportunity for outreach. At the same time, the literature cautions that religious and community platforms can also spread misinformation when not guided by accurate information. Building on these insights, another strong predictor of vaccine acceptance was caregivers’ recent contact with health providers. Those who had been contacted or visited by health workers were twice as likely to vaccinate their children. Female caregivers reported more frequent contact, reflecting their routine engagement in maternal and child health services. Similar gendered patterns have been observed in qualitative findings in Afar regions, where health posts are often perceived as female spaces, limiting men’s involvement. Additionally, the qualitative data reinforced the critical role of health workers, especially in pastoralist areas where outreach compensates for seasonal migration. However, inconsistent communication and impersonality during the COVID-19 pandemic undermined trust and reduced immunization uptake. House-to-house visits were effective only when health workers were trusted and culturally aligned; otherwise, skepticism persisted [42].

Turning to vaccine hesitancy, the study’s composite index found that over half of caregivers (51.1%) exhibited some degree of hesitancy, higher among males (56.3%) than females (48.4%). This contrasts with only 21.9% who self-reported hesitancy, highlighting likely underreporting due to social desirability bias. In addition, the considerably higher composite index value compared to the indicators based self-reported value suggested that many caregivers might not recognize or admit their own hesitancy, especially if they think hesitancy only means outright refusal. In reality, even doubts, fears, or delays can indicate hesitancy. This gap could also reflect a desire to give socially acceptable answers, or a lack of awareness about what counts as hesitancy. A similar pattern was observed in earlier studies in Ethiopia, where national surveys reported just 3–4% caregiver hesitancy, yet 56% of children experienced delayed vaccinations, indicating a gap between stated attitudes and actual behaviors [23]. The gendered discrepancy also aligns with regional evidence. In Nigeria, Galadima et al. (2021) found that male caregivers were over twice as likely to exhibit behavioral hesitancy compared to females, despite verbal agreement with vaccination [37]. The composite index in this study captured nuanced forms of hesitancy, such as doubt, delay, and ambivalence that are often missed by direct questioning. Male hesitancy was linked to lower involvement in immunization decisions and greater exposure to misinformation, especially in urban and conflict-affected areas. Qualitative findings described persistent myths circulating in male-dominated social spaces, while women’s frequent contact with health workers fostered vaccine confidence. Supporting this, multivariable analysis revealed that caregivers with strong family and religious leader support were significantly less likely to be hesitant. Women reported higher levels of such support (70.0% vs. 62.4% from religious leaders; 88.4% vs. 81.3% family support). Systematic reviews confirm these patterns, showing that family and religious support improve childhood vaccination uptake by 22–38% across low- and middle-income countries [9,37].

Qualitative data from this study also highlighted the importance of interpersonal trust, especially from spouses, mothers-in-law, and local leaders, in countering misinformation and encouraging vaccine uptake. In contrast, men’s lower reported support reflects structural barriers: Ethiopia’s Health Extension Program is predominantly staffed by women and primarily targets mothers, limiting male engagement. Another important predictor identified was fathers’ perceptions of vaccination responsibility. Fathers who view immunization as solely a woman’s duty were significantly more likely to have under-vaccinated children, highlighting how traditional gender norms contribute to vaccine hesitancy and delay timely immunization. In this study, most caregivers reported joint decision-making, yet many men still perceived vaccination as primarily the mother’s duty. Similarly, mothers were generally seen as the main caregivers responsible for immunization. This imbalance aligns with qualitative evidence from Uganda, where low male involvement and limited support during immunization visits hinder timely vaccine uptake [21].

The higher hesitancy observed in urban areas contrasts with earlier Ethiopian studies reporting greater rural hesitancy due to access barriers [43]. This shift likely reflects evolving information ecosystems where urban populations face greater exposure to conflicting messages and rumors, particularly regarding newer vaccines like COVID-19. Tailored urban interventions, including digital rumor surveillance and engagement of trusted male influencers, are critical to counteract misinformation and rebuild trust in these settings. The shift likely reflects changing urban information dynamics, where caregivers, particularly men, are more exposed to rumors and misinformation via social media and peer networks, fostering fears about vaccine safety and mistrust despite better service availability. Conversely, rural caregivers rely more on trusted local health workers and community networks for immunization information and encouragement [19]. These findings suggest access alone does not overcome distrust in urban settings. Additionally, employment status emerged as a significant factor influencing vaccine hesitancy, with employed caregivers demonstrating higher likelihood of hesitancy compared to their unemployed counterparts. Findings of the qualitative study linked this to time related constraints, exposed to rumors and misinformation via social media and peer networks and rigid work schedules, especially among men, limiting their participation in immunization activities. Women, notably in pastoralist communities, also faced access barriers when lacking partner support. Similar challenges have been documented in India and Mozambique, where work demands and inflexible clinic hours hinder timely vaccination [44,45].

Finally, education plays a crucial role in reducing vaccine hesitancy by improving knowledge, building confidence, and fostering critical thinking. However, qualitative findings revealed a paradox: some educated individuals, particularly urban men, were more likely to spread or believe misinformation, undermining vaccine confidence. Similar trends have been documented in South Africa and Nigeria, where higher education does not always guarantee vaccine acceptance due to conflicting information and distrust in authorities [46,47,48]. In contrast, in rural Ethiopian settings, caregivers with limited formal education but high trust in health workers and community programs often demonstrate strong vaccine acceptance [19]. These findings emphasize the need for context-specific education approaches that blend formal instruction with culturally grounded health communication.

Recent studies from low- and middle-income countries underscore the critical role of gender-transformative approaches in improving vaccine uptake [46,47,48]. For example, research in Nigeria and South Africa highlights how male engagement and addressing misinformation within male social networks can reduce hesitancy [46,48]. Similarly, implementation research in Mozambique and India demonstrates that flexible immunization services tailored to working caregivers improve timely vaccination [44,45]. These insights reinforce the need for context-specific, gender-responsive strategies in Ethiopia to sustain immunization gains.

### Policy Implications

To address these identified gender disparities in vaccine acceptance and hesitancy, Ethiopia’s Ministry of Health should integrate gender-transformative approaches into both the Expanded Program on Immunization (EPI) and the Health Extension Program (HEP). Efforts must explicitly promote shared parental responsibility, challenging entrenched perceptions that immunization is solely a mother’s role. Strengthening male engagement is critical and can be achieved through recruitment of male health workers and leveraging trusted platforms such as religious institutions, workplaces, and male-led community groups. These channels are essential for improving outreach and countering misinformation that disproportionately affects male caregivers. Collaboration with religious and community leaders should be enhanced by providing them with culturally sensitive, evidence-based communication tools.

Health literacy interventions must be context-specific, targeting men and urban populations via hybrid education models that combine formal instruction with community-based approaches. Immunization services in Ethiopia should better accommodate working caregivers by offering mobile vaccination units and organizing workplace vaccination campaigns, particularly in urban and peri-urban areas where caregivers often face scheduling challenges. Urban immunization strategies require particular attention, incorporating digital rumor surveillance and targeted social media campaigns that enlist trusted male influencers to combat misinformation prevalent in urban settings. In pastoralist and mobile communities, culturally competent outreach and flexible, family-centered services are vital to ensuring equitable access. Routine monitoring frameworks should include composite indices capable of capturing nuanced forms of hesitancy and disaggregated data by gender, location, and employment status to inform responsive interventions.

Mainstreaming gender in Health Extension Program training will better equip health workers to promote equitable decision-making and increase male involvement. To operationalize male engagement, the Ministry of Health should pilot recruitment and training of male health extension workers and community mobilizers who can effectively reach men in their social spaces. Integrating gender-sensitivity modules into Health Extension Program curricula will equip all health workers to address gender norms and promote shared parental responsibility. Leveraging workplaces, religious institutions, and male-led community groups as platforms for vaccine education can increase male caregiver involvement. Workplace vaccination campaigns and flexible service hours will accommodate employed caregivers’ schedules, particularly in urban areas. Monitoring frameworks should include gender-disaggregated indicators such as male caregiver participation rates and vaccine uptake to track progress toward gender equity in immunization. Finally, existing community structures such as the Health and Women’s Development Armies should be mobilized to actively engage both mothers and fathers, thereby closing gender gaps and advancing equitable vaccine coverage in Ethiopia. Such comprehensive, gender-transformative strategies are essential to sustaining immunization gains, improving resilience against health crises, and achieving universal vaccine equity.

This study generated valuable evidence on immunization but is limited by the potential for reporting bias due to self-reported vaccination status, acceptance, and hesitancy. Social desirability bias may have led caregivers to underreport vaccine hesitancy, as evidenced by the discrepancy between the composite hesitancy index (51.1%) and direct self-reported hesitancy (21.9%). Recall bias and interviewer effects may also have influenced responses, particularly for sensitive questions related to gender roles and decision-making. Future research should consider triangulating self-reports with vaccination card verification or health facility records to enhance data accuracy. Additionally, qualitative data collection in private settings aimed to minimize social desirability, but residual bias cannot be excluded.

## 5. Conclusions

This study demonstrates high vaccine acceptance among Ethiopian caregivers, driven by education and strong social and health system support. However, persistent vaccine hesitancy, particularly among male caregivers and urban residents, is influenced by traditional gender norms, misinformation, and employment-related barriers. Addressing these challenges requires gender-transformative, context-specific strategies that promote shared parental responsibility, engage men through trusted community platforms, and adapt immunization services to caregivers’ needs. Strengthening gender equity in immunization programs is essential to sustaining coverage gains and achieving universal vaccine equity in Ethiopia.

Future research should employ longitudinal designs to monitor changes in vaccine hesitancy and acceptance over time, particularly in response to gender-transformative interventions. Mixed-methods studies incorporating social network analysis could elucidate pathways of misinformation spread among male caregivers. Implementation research is needed to evaluate the feasibility and effectiveness of male engagement strategies and flexible immunization services in diverse Ethiopian contexts. Incorporating gender-disaggregated monitoring indicators will be essential to assess progress toward equitable vaccine coverage. While willingness to vaccination is high, hesitancy persists in the study regions. The study highlighted that expanding immunization across all age groups requires a shift toward gender-responsive strategies including integrating a gender perspective into the immunization programs that address traditional norms and misinformation.

### Limitations of the Study

This study has some limitations. Although the multi-stage sampling aimed to be robust, it may not fully represent all sub-populations, especially in conflict-affected areas like Tigray due to access challenges. Self-reported vaccination status could be biased by recall or social desirability. Employment status was used as a proxy for socioeconomic position, but direct income data were not collected, limiting economic insights. Regional differences were examined but showed no significant variation. Age was initially considered but excluded from the final model due to statistical criteria. Despite these limitations, the study provides valuable insights into gender and socioecological factors to guide more equitable immunization strategies.

## Figures and Tables

**Table 1:** Sociodemographic Characteristics of Study Participants, June 2025 (*n* = 980).

Variable	Category	Frequency (*n*)	Percentage (%)
Age group in years	18–34	645	65.8
	≥35	335	34.2
Marital Status	Married	904	92.2
	Divorced	38	3.9
	Others	38	3.9
Educational Status	Illiterate	185	18.9
	Primary	339	34.6
	Secondary	218	22.2
	Above secondary	238	24.3
Employment Status	Employed	276	28.2
	Unemployed	704	71.8
Religion	Muslim	535	54.6
	Orthodox	399	40.7
	Protestant	46	4.7
Total children	1	177	18.1
	2–3	493	50.3
	≥4	310	31.6
Children < 5 years	1	614	62.7
	≥2	366	37.3

**Table 2:** Ease of Access and Satisfaction with Childhood Vaccination Services (*n* = 980).

Indicator	Category	Female (*n* = 637)	Male (*n* = 343)	Total (*n* = 980)
Ease of paying for vaccination	Moderately/Very Easy	569 (89.3)	299 (87.2)	868 (88.6)
	Not at all easy	68 (10.7)	44 (12.8)	112 (11.4)
Know where to get child vaccinated	Yes	617 (96.9)	326 (95.0)	943 (96.2)
	No/Not sure	20 (3.1)	17 (5.0)	37 (3.8)
Ease of getting vaccination services	Moderately/Very Easy	624 (98.0)	337 (98.3)	961 (98.1)
	Not at all easy	13 (2.0)	6 (1.7)	19 (1.9)
Satisfaction with immunization services	Satisfied	609 (95.8)	324 (94.5)	933 (95.2)
	Unsatisfied	28 (4.2)	19 (5.5)	47 (4.8)

**Table 3:** Multivariable Logistic Regression Analysis of Factors Associated with Vaccine Acceptance (*n* = 979).

Variable	Category	Vaccine Acceptance Status		COR (95% CI)	AOR (95% CI)	*p*-Value
		Accepted	Not Accepted			
Education Level	Illiterate (ref)	169	16	1.00	1.00	–
	Primary school	319	20	0.53 (0.27–1.05)	0.48 (0.32–0.72)	0.000 ***
	Secondary	209	9	0.33 (0.15–0.71)	0.33 (0.21–0.52)	0.000 ***
	Above Secondary	219	19	0.47 (0.23–0.95)	0.47 (0.29–0.79)	0.004 **
Marital Status	Married (ref)	850	54	1.00	1.00	–
	Unmarried	66	10	0.42 (0.20–0.90)	1.33 (0.79–2.25)	0.288
Family Support Vaccine	No (ref)	118	20	1.00	1.00	–
	Yes	798	44	0.33 (0.20–0.56)	0.44 (0.28–0.70)	0.001 **
Religion Supports Vaccine	No (ref)	292	28	1.00	1.00	–
	Yes	624	36	0.59 (0.36–0.98)	0.58 (0.42–0.80)	0.001 **
Health Worker Recommended	Yes (ref)	833	44	1.00	1.00	–
	No	83	20	0.36 (0.22–0.58)	1.15 (0.81–1.64)	0.432
Health Worker Contacted	No (ref)	215	30	2.88 (1.72–4.81)	1.00	–
	Yes	701	34	2.88 (1.72–4.81)	2.01 (1.07–3.76)	0.030 *
Know Where to Immunize	Doesn’t Know (ref)	29	8	1.00	1.00	–
	Knows Where	887	56	4.29 (1.86–9.91)	0.49 (0.23–1.06)	0.068
Attitude Women Resp	Disagree (ref)	322	15	1.00	1.00	–
	Agree	593	49	0.56 (0.31–1.02)	0.74 (0.38–1.42)	0.364

Significance levels: *p* < 0.05 (*); *p* < 0.01 (**); *p* < 0.001 (***).

**Table 4:** Multivariable Logistic Regression of Factors Associated with Vaccine Hesitancy Among 979 Participants.

Variables	Category	Vaccine Hesitancy Status		COR (95% CI)	A OR (95% CI)	*p*-Value
		Not Hesitant	Hesitant			
Sex of Respondent	Female (ref)	329	308	1.00	1.00	–
	Male	150	193	1.37 (1.06–1.79)	0.73 (0.41–1.33)	0.304
Attitude Toward Women	Disagree (ref)	188	149	1.00	1.00	–
	Agree	291	351	1.52 (1.17–1.98)	1.69 (1.25–2.28)	0.001 **
Education Level	Illiterate (ref)	78	107	1.00	1.00	–
	Primary school	181	158	0.58 (0.41–0.81)	0.88 (0.54–1.44)	0.613
	Secondary school	125	93	0.49 (0.34–0.72)	0.53 (0.31–0.90)	0.019 *
	Above secondary	95	143	—	Interaction term used	—
Residence	Rural (ref)	272	220	1.00	1.00	–
	Urban	207	281	1.68 (1.30–2.16)	1.62 (1.21–2.16)	0.001 **
Employment Status	Employed (ref)	105	171	1.00	1.00	–
	Unemployed	374	330	0.54 (0.41–0.72)	0.59 (0.40–0.88)	0.009 **
Family Support	No (ref)	33	105	1.00	1.00	–
	Yes	446	396	0.28 (0.18–0.42)	0.25 (0.09–0.71)	0.009 **
Religious Support	No (ref)	113	207	1.00	1.00	–
	Yes	366	294	0.44 (0.33–0.58)	0.50 (0.36–0.71)	0.000 ***
Health Worker Recommended	No (ref)	29	74	0.37 (0.24–0.58)	0.40 (0.15–1.08)	0.070
	Yes	450	427	1.00	1.00	–
Health Worker Contact	No (ref)	97	148	1.00	1.00	–
	Yes	382	353	0.61 (0.45–0.81)	0.93 (0.66–1.32)	0.696
Knows Where to Immunize	Doesn’t Know (ref)	11	26	1.00	1.00	–
	Knows Where	468	475	0.43 (0.21–0.88)	0.48 (0.22–1.01)	0.052

Significance levels: *p* < 0.05 (*), *p* < 0.01 (**), *p* < 0.001 (***), marginally significant (*p* ≈ 0.05).

## Data Availability

The data presented in this study are available on request from the corresponding author due to privacy reasons.
